# Investigating the Effects of Time and Temperature on the Growth of *Escherichia coli* O157:H7 and *Listeria monocytogenes* in Raw Cow’s Milk Based on Simulated Consumer Food Handling Practices

**DOI:** 10.3390/ijerph16152691

**Published:** 2019-07-28

**Authors:** Roselyn M. Leclair, Sarah K. McLean, Louise A. Dunn, Denny Meyer, Enzo A. Palombo

**Affiliations:** 1 Department of Chemistry and Biotechnology, Swinburne University of Technology, Hawthorn, Victoria 3122, Australia; 2 Department of Statistics, Data Science and Epidemiology, Swinburne University of Technology, Hawthorn, Victoria 3122, Australia

**Keywords:** raw milk, consumer food handling practices, *Escherichia coli* O157:H7, *L. monocytogenes*

## Abstract

Consumption of raw cow’s milk (RCM) is increasing in popularity in developed countries despite the associated foodborne disease risks. While previous research has focused on consumer motivations for drinking RCM, there is limited research on how consumer handling practices may impact the microbiological safety of RCM. In this study, consumer handling practices associated with transport, storage, and freezing and thawing were simulated to investigate the impact of time and temperature variables on the concentrations of either *Escherichia coli* O157:H7 or *Listeria monocytogenes* in RCM. We found that the type of storage during simulated transport had a large (η^2^ = 0.70) and significant (*p* < 0.001) effect on both pathogens. The refrigeration temperature also had a large (η^2^ = 0.43) and significant (*p* < 0.001) effect on both pathogens during refrigerated storage. The interaction between pathogen species and initial pathogen inoculum level had a large (η^2^ = 0.20) and significant (*p* = 0.012) effect on the concentration of the pathogens during ambient temperature storage. We found that freezing and thawing practices did not have a significant effect on the pathogens (*p* > 0.05). However, we were able to recover *L. monocytogenes*, but not *E. coli* O157:H7, from RCM after freezing for 365 days. The results from this study highlight that consumer transport and storage practices can have significant effects on the growth of *E. coli* O157:H7 and *L. monocytogenes* in RCM. Consumer food handling practices should be considered when developing public health strategies aimed at reducing the risks of RCM consumption.

## 1. Introduction

Raw milk can be defined as milk that has not been pasteurised or undergone treatment of an equivalent effect [1,2]. In developed countries, cow’s milk is commonly pasteurised in order to destroy pathogenic bacteria [3]. However, there appears to be a growing trend towards the consumption of raw cow’s milk (RCM) in these countries [4]. Studies have found that consumer motivations for drinking RCM include enhanced nutritional qualities, taste and potential health benefits [5,6]. Additionally, naturally occurring bacteria in raw milk, such as lactic acid bacteria (LAB), have been shown to have an antimicrobial effect on pathogens [7]. However, the associated foodborne disease risks of RCM consumption are well established [3,8].

Bacterial pathogens such as *Escherichia coli* O157:H7 and *Listeria monocytogenes* have been associated with raw milk associated outbreaks in Europe, the UK and the US [8,9,10,11,12,13]. The infective dose of *E. coli* O157:H7 is speculated to be low (10 to 100 cells), and can result in serious illnesses, particularly in children and other vulnerable populations [14]. Therefore, according to Australian microbiological criteria, the presence of pathogenic *E. coli* in foods is considered unsatisfactory [15]. *L. monocytogenes* can also cause serious illness in vulnerable groups and has a high case-fatality rate [14]. In ready-to-eat (RTE) foods where contamination can occur but the growth of *L. monocytogenes* will not occur, such as frozen ice cream, Australian standards specify a limit of 100 CFU/g [15]. However, the absence of *L. monocytogenes* in 25 g of the product is specified for foods that can support its growth [15]. *L. monocytogenes* has the ability to grow at refrigeration temperatures [16] and studies have reported varied prevalence between 1.0% and 12.6% in bulk tank milk [17,18,19].

Pathogenic bacteria may be present in raw milk or introduced at various stages throughout the food supply chain [20]. Public health strategies, such as the Hazard Analysis Critical Control Point (HACCP) system, aim to reduce the microbial risks associated with raw milk along the food supply chain, prior to the consumer phase [21,22]. However, research suggests that consumer food handling can contribute to foodborne disease and negate food safety practices during production and processing [23,24,25]. Poor consumer food handling may result in time/temperature abuse in refrigerated foods such as milk [26] and aid the growth of bacteria.

Recommendations for consumers on the handling of raw milk are limited, other than to avoid consuming raw milk [27,28]. Some guidelines recommend consumers keep raw milk chilled while transporting it home, storing raw milk in the coldest part of the refrigerator, drinking raw milk by the use-by-date and to boil raw milk prior to consumption [29]. Although the practice of boiling raw milk prior to consumption would eliminate the microbiological risks associated with raw milk, this would minimise the appeal for consumers desiring to consume raw, unpasteurised, milk [30]. In fact, a study in Italy found that, despite recommendations, 43% of consumers did not boil raw milk before consumption [31]. Additionally, studies have suggested that consumers freeze raw milk for later use [30,32], however freeze/thaw practices are not included in the available guidelines for handling raw milk.

Therefore, consumer behaviour is an important consideration when designing public health strategies to reduce the risks associated with RCM consumption. Effective strategies need to have a strong evidence base to inform policymakers and contribute to the data required for risk assessments [33].

This study aimed to investigate how time and temperature variables associated with the food handling practices of consumers affect the growth of *E. coli* O157:H7 and *L. monocytogenes* in RCM prior to consumption.

## 2. Materials and Methods

### 2.1. Sample Collection

RCM samples were collected from farm bulk tanks by laboratory staff from two dairy manufacturers in Victoria, Australia. Sample batches were collected between 2015 and 2017 in 100–150 mL sample volumes, and each sample was collected from a different bulk tank. Samples were transported to Swinburne University on ice in an insulated container within one hour of collection and tested on the same day.

### 2.2. Challenge Testing of Escherichia coli O157:H7 and Listeria monocytogenes in Raw Cow’s Milk

Streptomycin-resistant strains of *Escherichia coli* O157:H7 (EDL 933 ATCC 700927), hereafter denoted as *E. coli* O157:H7 Str^R^, or *Listeria monocytogenes* (ACM 98), hereafter denoted as *L. monocytogenes* Str^R^, were used to simulate the growth of pathogenic bacteria in RCM samples. Streptomycin-resistant strains were used to enable isolation of the pathogens in the presence of background bacteria in RCM samples [34]. Two inoculum levels of *E. coli* O157:H7 Str^R^ or *L. monocytogenes* Str^R^, approximately 10^6^ and 10^2^ CFU/mL, were used to investigate growth in RCM with high and low contamination loads, respectively. Although the low contamination load used is above the threshold allowed in foods for both pathogens according to Australian standards [35], this concentration was considered appropriate for the challenge testing because a lower bacterial concentration is below the reliable limit of detection [36]. Additionally, similar low and high inoculation levels have been used to evaluate the behaviour of these pathogens in raw milk [31].

Bacterial suspensions were prepared in 0.85% saline and adjusted to a density equivalent to the 0.5 McFarland turbidity standard using a colorimeter. Solutions were diluted to an approximate concentration of either 10^3^ CFU/mL or 10^7^ CFU/mL and then 1 mL of bacterial solution was added to 9 mL of RCM to achieve the desired final concentration. Inoculated RCM samples were then used in consumer handling practice simulations.

An aliquot of RCM containing no added *E. coli* O157:H7 Str^R^ or *L. monocytogenes* Str^R^ was removed from each sample at baseline and plated as described in Section 2.4.1, with no growth confirming that no streptomycin-resistant bacteria were naturally present at or above the level of detection.

### 2.3. Consumer RCM Handling Practice Simulations

Inoculated RCM samples were subjected to laboratory conditions simulating consumer RCM handling practices associated with transport, storage and freezing and thawing practices as described below.

#### 2.3.1. Transport Simulation

Inoculated RCM samples were stored: (1) In an insulated storage box (Coleman^®^ 4.7 L capacity) with an ice brick (Esky^®^ 350 mL at −18 °C) or (2) in the insulated storage box without an ice brick or (3) without an insulated storage box. Samples stored in either 1, 2 or 3 as outlined above, were placed inside a shaker incubator at 100 rpm to simulate movement inside a motor vehicle for 30 min at 20 °C, 30 °C or 40 °C. For each unique treatment effect combination (storage type and shaker incubation temperature) samples (*n* = 4) were prepared in triplicate. A 100 μL aliquot was removed from the samples at 0, 5, 15 and 30 min for enumeration as described in Section 2.4.1.

#### 2.3.2. Storage Simulations

##### Refrigerated Storage

Inoculated RCM samples prepared in triplicate were either stored in a Thermoline^®^ laboratory refrigerator at a recommended refrigeration temperature of 4 °C (*n* **=** 27) or, to represent a mild and moderate temperature abuse, stored in a refrigerated incubator at 8 °C (*n* **=** 24) or 15 °C (*n* **=** 20), respectively, for 5 days. A 100 μL aliquot was removed from each sample between day 0 and day 5 for enumeration as described in Section 2.4.1.

##### Ambient Temperature Storage

To investigate the behaviour of the pathogens during the natural souring of raw milk when stored at ambient temperatures, each inoculated RCM sample (*n* = 29) was prepared in triplicate and incubated for 10 days at 22 °C. A 100 μL aliquot was removed each day between day 0 and day 5 and then at day 9 and 10 for enumeration as described in Section 2.4.1. Due to the role that LAB has in the natural souring of raw milk [7,37], LAB were also enumerated for this simulation as described in Section 2.4.2. The pH was monitored using a pH meter (Cheetah^®^ PHS-3C, ±0.01), calibrated before each use.

#### 2.3.3. Freeze/Thaw Simulation

Inoculated RCM samples prepared in triplicate were frozen at −18 °C either immediately following inoculation (*n* = 26) or after four days of incubation at 4 °C (*n* = 28). Samples were removed from the freezer after 7, 14, 30, 60, 90 or 365 days and thawed overnight at 4 °C or incubated at 22 °C until visibly thawed to simulate refrigerated and ambient temperature thawing, respectively. A 100 μL aliquot was removed after thawing for enumeration as described in Section 2.4.1.

### 2.4. Determination of Viable Counts

#### 2.4.1. Enumeration of Pathogens

Aliquots of RCM samples removed during food handling practice simulations were serially diluted in 0.1% Buffered Peptone Water (BPW) up to 10^8^. Dilutions were plated in triplicate onto Brain Heart Infusion (BHI) agar supplemented with 300 μg/mL streptomycin. Plates were incubated at 37 °C for 24 h [34]. Plates with less than 300 colonies were selected for manual enumeration and the final number converted to logarithm base 10 (Log_10_ CFU/mL).

#### 2.4.2. Enumeration of LAB

Aliquots of RCM samples removed during ambient temperature storage simulations were serially diluted in 0.1% BPW and dilutions up to 10^8^ were plated in triplicate onto De Man, Rogosa and Sharpe (MRS) agar. MRS plates were incubated in an atmosphere of 5% CO_2_ at 37 °C for 24 h, or for 48 h if no characteristic LAB growth was observed after 24 h. Plates with less than 300 colonies were selected for manual enumeration and the final number converted to Log_10_ CFU/mL.

### 2.5. Statistical Analyses

Statistical analyses were conducted using IBM Statistical Package for the Social Sciences (SPSS) Version 24. For each simulation, a General Linear Model (GLM) analysis with repeated measures over time was used to analyse the significance of the effects of the independent variables (IVs) on the pathogens (dependent variable, DV) over time. A *p*-value < 0.05 was considered statistically significant. Main effects and two-way interaction effects were tested and significant results were visualised using Estimated Marginal Means (EMMeans) plots, controlling for factors not shown in the plots. Marginal means are the estimated mean values when assuming average values for all other variables in the model [38]. Measure of effect size was determined as small (η^2^ < 0.06), moderate (η^2^ between 0.06 and 0.14) or large (η^2^ > 0.14) [39].

For the transport simulation, a General Linear Model (GLM) analysis with repeated measures was used to analyse the significance of the effects of the IVs (pathogen species, pathogen inoculum level, storage type and incubation temperature plus shaking) on the pathogens over time.

For the refrigerated storage simulation, a GLM analysis with repeated measures was used to analyse the significance of the effects of the IVs (refrigeration temperature, pathogen species and pathogen inoculum level) on the pathogens over time.

For the ambient temperature storage simulation, a GLM analysis with repeated measures was used to analyse the significance of the effects of the IVs (pathogen species and pathogen inoculum level) on the pathogens over time.

The Greenhouse—Geisser adjustment was applied when the assumption of sphericity was rejected for the above simulations.

For the freeze/thaw simulation, a GLM analysis was used to analyse the significance of the effects of the IVs (pre-freeze storage time, thawing method, days frozen, pathogen species and pathogen inoculum level) on the pathogens while controlling for initial pathogen concentration levels.

## 3. Results

### 3.1. Transport Simulation

The type of storage, the incubation temperature plus shaking and the pathogen species all had large (η^2^ > 0.14) and significant (*p* < 0.05) effects on the pathogens over 30 min (Table S1). The interaction between storage type and incubation temperature plus shaking also had a large (η^2^ = 0.43) and significant (*p* = 0.03) effect.

**Figure 1:**
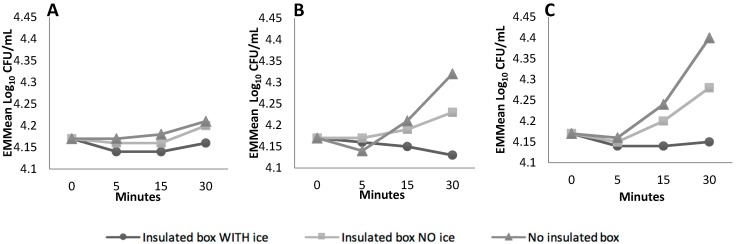
Estimated Marginal Means (EMM)eans of pathogen concentration under various storage types at 20 °C (**A**), 30 °C (**B**) and 40 °C (**C**) over 30 min, statistically controlling for pathogen species and pathogen inoculum level factors.

**Figure 2:**
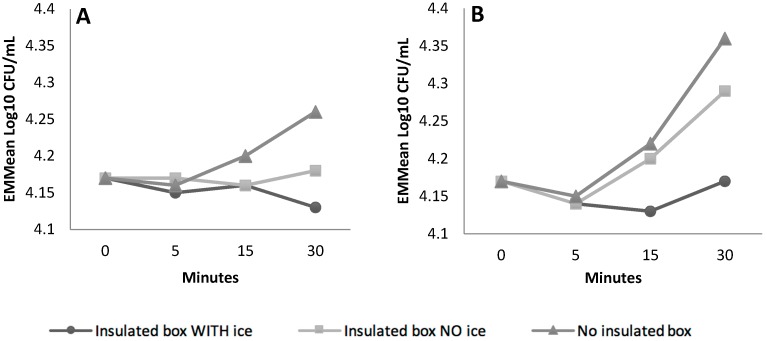
EMMeans of *E. coli* O157:H7 (**A**) and *L. monocytogenes* (**B**) concentrations under various storage types over 30 min, statistically controlling for shaker incubation temperature and pathogen inoculum level factors.

The EMMeans plots show an increase in pathogen concentration across all incubation temperatures and storage types, except for an insulated box containing an ice brick (Figure 1) where the concentration of *E. coli* O157:H7 Str^R^ decreased and *L. monocytogenes* Str^R^ was restored to initial baseline concentration after 30 min (Figure 2).

Initial pathogen inoculum level did not have a significant effect (*p* > 0.05) on the concentration of the pathogens over time.

### 3.2. Storage Simulations

#### 3.2.1. Refrigerated Storage Simulation

**Figure 3:**
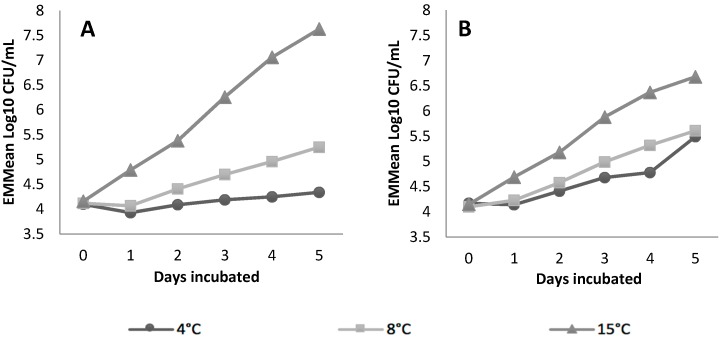
EMMeans of *E. coli* O157:H7 (**A**) and *L. monocytogenes* (**B**) concentrations at 4 °C, 8 °C and 15 °C over 5 days, statistically controlling for pathogen inoculum level.

Refrigeration temperature had a large (η^2^ = 0.43) and significant (*p* < 0.001) effect on the pathogens and the interaction between the refrigeration temperature and the pathogen species had a moderate (η^2^ = 0.12) and significant (*p* = 0.009) effect (Table S2). The EMMeans plots show that the concentration of *L. monocytogenes* Str^R^ increased more rapidly over 5 days than *E. coli* O157:H7 Str^R^ at 4 °C and 8 °C, but not at 15 °C (Figure 3).

Initial pathogen inoculum level did not have a significant effect (*p* > 0.05) on the concentration of the pathogens over time.

#### 3.2.2. Ambient Temperature Storage Simulation

**Figure 4:**
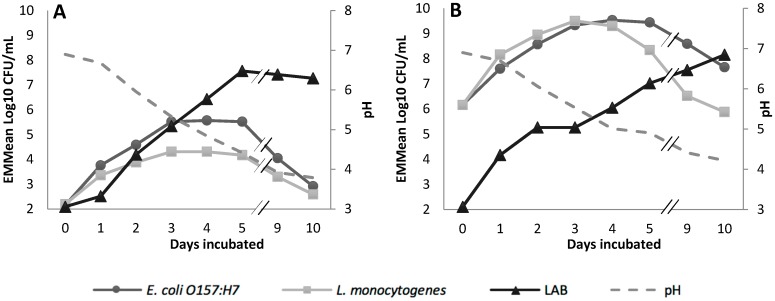
EMMeans of *E. coli* O157:H7, *L. monocytogenes,* lactic acid bacteria (LAB) and pH at a lower 10^2^ (**A**) and higher 10^6^ (**B**) pathogen inoculum level over 10 days at 22 °C.

The interaction between pathogen species and pathogen inoculum level had a large (η^2^ = 0.20) and significant (*p* = 0.012) effect on the pathogens (Table S3). *E. coli* O157:H7 Str^R^ increased more rapidly when inoculated at a lower concentration. Both pathogens declined close to baseline levels at the lower inoculum level. However the EMMeans of *E. coli* O157:H7 Str^R^ viable count was higher than *L. monocytogenes* Str^R^ over 10 days at the higher concentration (Figure 4).

The EMMeans of the LAB viable count increased by 5.5 log CFU/mL over 5 days at the lower pathogen inoculum level before reaching a stationary phase. At the higher pathogen inoculum level, the LAB increased by 4.9 log CFU/mL over 5 days, however no stationary phase was observed at day 9 or 10. The pH decreased by 3.1 and 2.7 at the low and high pathogen inoculum levels, respectively.

### 3.3. Freeze/Thaw Simulation

**Figure 5:**
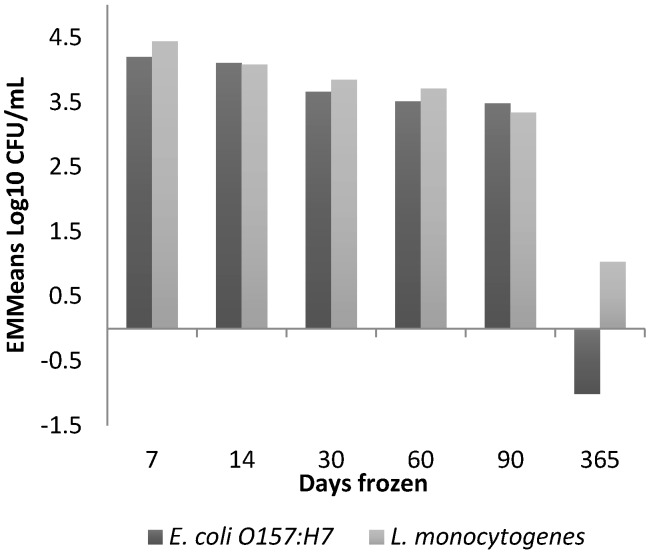
EMMeans of *E. coli* O157:H7 and *L. monocytogenes* concentrations after freeze/thaw effects and days of frozen storage. Adjusted for the covariate of mean pathogen concentration at baseline.

The pathogen species and the duration of frozen storage had a significant effect on the viability of both pathogens in RCM (Table S4). The interaction between the pathogen species and the duration of frozen storage had a large (η^2^ = 0.27) and significant (*p* = 0.032) effect. While *L. monocytogenes* Str^R^ was recovered following the freeze/thaw process after 365 days, the EMMeans of *E. coli* O157:H7 Str^R^ indicate that this pathogen did not survive over this time (Figure 5). The pre-freeze duration (immediately frozen vs. 4 day delay), thawing methods (overnight at 4 °C or incubated at 22 °C until thawed) and pathogen inoculum level did not have a significant effect (*p* > 0.05) on the pathogens. After removing the 365 day parameter from the GLM analysis, we found no significant outcomes across all factors.

## 4. Discussion

Consumer food handling practices related to transport, refrigeration and storage can impact overall food safety. While consumer guidelines recommend transporting chilled foods in an insulated container, especially in warmer weather, studies have indicated that this is not routinely practiced by consumers [40,41]. This is a concern as the concentration of the pathogens in this study was best inhibited when using an insulated storage container, particularly at the higher incubation temperatures. Inclusion of an ice-brick was shown to inhibit but not eliminate *E. coli* O157:H7 Str^R^. These results highlight the importance of consumer guidelines that include the recommendation of an ice brick or similar cooling object during transport to prevent bacterial growth.

Previous studies have demonstrated that domestic refrigerators often operate above recommended temperature, that consumers are unaware of the temperature status of their refrigerators and that milk is not commonly stored in the coldest area of the refrigerator [26,42,43,44]. In this study, the concentration of both pathogens increased more rapidly under mild (8 °C) and moderate (15 °C) temperature abuse conditions compared to the recommended refrigeration temperature of 4 °C. Although, by day 5, the difference in the EMMeans of the *L. monocytogenes* Str^R^ stored at 4 °C and 8 °C was only 0.12 log CFU/mL. The growth of *L. monocytogenes* in refrigerated foods is well established due to its psychrotrophic nature and our findings support previous research demonstrating the growth of *L. monocytogenes* in refrigerated RCM [16,44,45]. The findings demonstrate that the refrigerated storage of RCM can contribute to pathogen growth over time, even at recommended refrigeration temperatures.

Naturally soured or spontaneously fermented raw milk products are usually stored at ambient temperature for several days before consumption [46,47]. In the current study, the pathogens were inhibited between days 3 and 5, while LAB increased and pH decreased. The inhibition of the pathogens may have been due to the acidic conditions or the antimicrobial effect of LAB in fermented dairy products [7,47]. Although the pathogens used in this study were inhibited, other studies have shown these pathogens can exhibit acid tolerance and have been implicated in outbreaks associated with acidic foods [48,49]. The initial pathogen inoculum levels were only a significant factor in the ambient temperature simulation, indicating that different levels of contamination can impact growth and survival of pathogens in raw milk stored at ambient temperatures [50]. While ambient temperature storage of RCM could increase the risk of foodborne illness, soured milk can be subjected to further processing which may inhibit or eliminate pathogens in the final product [50,51]. Further information on consumer behaviours associated with the natural souring of RCM is needed to inform risk assessments on this practice.

Consumer freezing and thawing practices may result in temperature abuse of foods which can contribute to microbial growth [52]. Freezer storage recommendations for RTE foods are to ensure food is frozen immediately to maintain temperature control [53]. Studies have demonstrated domestic refrigerators are not regularly maintained at recommended temperatures [26,42,43], therefore RCM stored for several days before freezing can contribute to further time/temperature abuse. Although there was no statistical significance associated with the pre-freeze duration (immediately frozen vs. 4-day delay), long term (365 days) frozen storage had a significant effect on the viability of *E. coli* O157:H7 Str^R^. A decrease in both pathogens was observed, although neither pathogen was significantly affected by frozen storage up to 90 days. However, other studies have found a significant decrease in *E. coli* with increasing length of frozen storage in milk [54,55].

Previous studies have found that consumers often thaw potentially hazardous foods at room temperature [40] despite recommendations to thaw foods overnight in the refrigerator [53]. There was no statistically significant outcome associated with the thawing methods used in this study, however previous research has found that different thawing methods can have a significant effect on the recovery of bacteria in milk [56].

Furthermore, this study investigated the effect of consumer food handling practices based on individual stages within the consumer phase of the food supply chain, prior to consumption. In practice, however, consumers would continually handle and consume RCM over time and the cumulative effect of potential time and temperature abuse would likely affect the overall quality and safety of the product.

## 5. Limitations

The study aimed to simulate RCM consumer food handling practices, however we acknowledge that the sample volume (10 mL) may not reflect the larger volumes consumers are likely to use and which may have impacted the rate in which samples reached various temperature parameters. The simulations could be enhanced by representative volumes of RCM.

## 6. Conclusions

The results highlighted that using an ice brick with an insulated storage container during transport and the refrigerated storage of raw milk at 4 °C were critical for controlling the concentrations of the pathogenic bacteria tested in this study in RCM. Further research should also consider strain variations of the selected pathogens and the cumulative effect of time and temperature abuse throughout the consumer phase that can result in microbial growth. Public health strategies to reduce the risks associated with RCM could be improved by highlighting the importance of consumer handling practices between sourcing and consumption.

## Supplementary Materials

The following are available online at https://www.mdpi.com/1660-4601/16/15/2691/s1.
