# Supplementary material for: Investigating the Effects of Time and Temperature on the Growth of Escherichia coli O157:H7 and Listeria monocytogenes in Raw Cow’s Milk Based on Simulated Consumer Food Handling Practices
Source: Int J Environ Res Public Health. 2019 Jul 28;16(15):2691. doi: 10.3390/ijerph16152691 (PMC6696089; doi:10.3390/ijerph16152691)
Supplement: Supplementary file 1 [file ijerph-16-02691-s001.pdf]

Supplementary Table S1. Interaction significance in mean pathogen concentrations over 30 minutes between storage type, shaker incubation temperature and pathogen species factors.

Factor	Levels	Marginal Means (Error)
**Storage type**		
$F(3.3, 34.9) = 24.9$	Insulated box with ice brick	4.15 (.005)
$p < 0.001$	Insulated box no ice brick	4.19 (.005)
$\eta^2 = .70$	No insulated box	4.21 (.005)
**Shaker incubation temperature**		
$F(3.3, 34.9) = 8.0$	20 °C	4.17 (.005)
$p < 0.001$	30 °C	4.18 (.005)
$\eta^2 = .43$	40 °C	4.20 (.005)
**Pathogen species**		
$F(1.7, 34.9) = 27.6$	*E. coli* O157:H7	4.17 (.004)
$p < 0.001$	*L. monocytogenes*	4.19 (.004)
$\eta^2 = .57$		
**Storage type * Shaker incubation temperature**		
$F(6.6, 34.9) = 3.9$		
$p = 0.03$	Insulated box with ice brick (20 °C)	4.15 (.008)
$\eta^2 = .4$	Insulated box with ice brick (30 °C)	4.15 (.008)
	Insulated box with ice brick (40 °C)	4.15 (.008)
	Insulated box no ice brick (20 °C)	4.17 (.008)
	Insulated box no ice brick (30 °C)	4.19 (.008)
	Insulated box no ice brick (40 °C)	4.20 (.008)
	No insualted box (20 °C)	4.18 (.008)
	No insualted box (30 °C)	4.20 (.008)
	No insulated box (40 °C)	4.24 (.008)

Supplementary Table S2. Interaction significance in mean pathogen concentrations over 5 days between refrigeration temperature and pathogen species factors.

Factor	Levels	Marginal Means (Error)
**Refrigeration temperature**		
$F(2.8, 85.0) = 22.8$	4 °C	4.38 (.089)
$p < 0.001$	8 °C	4.70 (.096)
$\eta^2 = .43$	15 °C	5.69 (.100)
**Pathogen species**		
$F(1.5, 85.0) = 0.3$	*E. coli* O157:H7	5.02 (.310)
$p > 0.05$	*L. monocytogenes*	4.87 (.508)
$\eta^2 = .01$		
**Refrigeration temperature * Pathogen species**		
$F(2.8, 85.0) = 3.9$		
$p = 0.009$	4 °C (*E. coli* O157:H7)	4.15 (.090)
$\eta^2 = .12$	4 °C (*L. monocytogenes)*	4.61 (.153)
	8 °C (*E. coli* O157:H7)	4.59 (.095)
	8 °C (*L. monocytogenes)*	4.81 (.169)
	15 °C (*E. coli* O157:H7)	5.88 (.107)
	15 °C (*L. monocytogenes)*	5.49 (.169)

Supplementary Table S3. Interaction significance in mean pathogen concentrations at ambient temperature (22 °C) storage over 10 days between pathogen species and pathogen inoculum level factors.

Factor	Levels	Marginal Means (Error)
**Pathogen species**		
$F(1.3, 33.1) = 6.6$	*E. coli* O157:H7	6.31 (.105)
$p = 0.009$	*L. monocytogenes*	5.68 (.145)
$\eta^2 = .21$		
**Pathogen inoculum level**		
$F(1.3, 33.1) = 1.4$	10^2 CFU/mL	3.89 (.125)
$p > 0.05$	10^6 CFU/mL	8.11 (.128)
$\eta^2 = .05$		

**Pathogen species * Pathogen inoculum level**		
$F(1.3, 33.1) = 6.1$		
$p = 0.012$	10^2 CFU/mL (*E. coli* O157:H7)	4.26 (.145)
$\eta^2 = .20$	10^2 CFU/mL *(L. monocytogenes)*	3.52 (.205)
	10^6 CFU/mL (*E. coli* O157:H7)	8.36 (.153)
	10^6 CFU/mL *(L. monocytogenes)*	7.85 (.205)

Supplmentary Table S4. Interaction significance in mean pathogen concentrations after freeze/thaw effects between pathogen species and days of frozen storage, controlling for baseline pathogen concentration.

**Factor**	**Levels**	**Marginal Means (Error)**
**Pathogen species**		
$F(1.0, 38.0) = 5.7$	*E. coli* O157:H7	2.99 (.146)
$p = 0.022$	*L. monocytogenes*	3.41 (.111)
$\eta^2 = .13$		
**Days frozen**		
$F(5.0, 38.0) = 49.9$	7 days	4.32 (.175)
$p < 0.001$	14 days	4.10 (.180)
$\eta^2 = .87$	30 days	3.76 (.170)
	60 days	3.61 (.189)
	90 days	3.41 (.313)
	365 days	0.01 (.247)
**Pathogen species * Days frozen**		
$F(5.0, 38.0) = 2.8$	7 days (*E. coli* O157:H7)	4.20 (0.241)
$p = 0.032$	7 days *(L. monocytogenes)*	4.44 (0.250)
$\eta^2 = .27$	14 days (*E. coli* O157:H7)	4.11 (0.235)
	14 days *(L. monocytogenes)*	4.08 (0.272)
	30 days (*E. coli* O157:H7)	3.66 (.199)
	30 days *(L. monocytogenes)*	3.85 (.280)
	60 days (*E. coli* O157:H7)	3.51 (.263)
	60 days *(L. monocytogenes)*	3.71 (.259)
	90 days (*E. coli* O157:H7)	3.48 (.560)
	90 days *(L. monocytogenes)*	3.34 (.286)
	365 days (*E. coli* O157:H7)	-1.01 (.411)
	365 days *(L. monocytogenes)*	1.03 (.277)
